# ASIC1a-Dependent Potentiation of Acid-Sensing Ion Channel Currents by Cyanide

**DOI:** 10.3390/biom15040479

**Published:** 2025-03-25

**Authors:** Qian Jiang, Felix Yang, Amber Sun, Yuyang Chu, Joseph Cascone, Dylan Glaser, Xiang-Ping Chu

**Affiliations:** 1 Department of Biomedical Sciences, School of Medicine, University of Missouri-Kansas City, Kansas City, MO 64108, USA; qjiang@umkc.edu (Q.J.); felixyang@umkc.edu (F.Y.); awsun@umkc.edu (A.S.); jvcgwc@umkc.edu (J.C.); dsg8hy@umkc.edu (D.G.); 2 Department of Orthopedic Surgery, Detroit Medical Center, School of Medicine, Wayne State University, Detroit, MI 48201, USA; ychu2@dmc.org

**Keywords:** acid-sensing ion channels, cyanide, zinc, point mutation, patch-clamp recording, cultured cortical neuron, gene transfection

## Abstract

Cyanide (CN) is a potent, fast-acting toxicant that impacts endogenous biomolecules in the nervous system, including acid-sensing ion channels (ASICs), which play a vital role in various neurological and psychological conditions. Here, we demonstrate that CN rapidly potentiates ASIC currents in cultured mouse cortical neurons in a dose-dependent manner while causing a leftward shift in the pH dose–response curve. Notably, this potentiation was unaffected by a 30-min CN treatment or the presence of ATP in the recording pipette. Further investigations into the role of zinc revealed that TPEN, a high-affinity zinc chelator, did not enhance ASIC currents following CN pretreatment, nor did CN influence the potentiation of ASIC currents induced by TPEN. Low-affinity zinc blocked the potentiation of ASIC currents by CN. CN potentiated ASIC currents in cortical neurons from ASIC2 but not from ASIC1a knockout mice. In experiments with CHO cells expressing homomeric ASIC1a and heteromeric ASIC1a/2, CN potentiated ASIC1a currents but had no effect on homomeric ASIC1b, ASIC2a, or ASIC3 channels. Mutating lysine 133 (K133) to arginine (R) in the extracellular domain of ASIC1a abolished CN’s effect, suggesting that CN potentiates ASIC1a currents primarily via high-affinity zinc binding, with K133 being critical for this modulation.

## 1. Introduction

Acid-sensing ion channels (ASICs) are a family of ion channels that are primarily activated by extracellular acidification [1]. These channels are highly expressed in the central nervous system and play a pivotal role in maintaining cellular homeostasis, regulating neuronal excitability, and contributing to sensory processes such as pain and touch [2,3]. ASICs are particularly important for synaptic function, where they influence synaptic plasticity and contribute to neurophysiological processes like learning and memory [4,5]. They are also involved in the detection of environmental pH changes, such as those that occur during ischemic events, where the blood supply to brain tissue is compromised, leading to local acidification [6]. ASICs have been implicated in a variety of neurological and psychological conditions. These include ischemic stroke [6,7], traumatic brain injury [8,9], epilepsy [10,11], chronic pain [12,13], and drug addiction [4,14]. During pathological conditions, such as cerebral ischemia, the acidification of the extracellular environment leads to the activation of ASICs, which contributes to neuronal depolarization, excitotoxicity, and subsequent neuronal damage [6,7]. Thus, while ASICs have vital roles in normal physiological processes, their dysregulation or overactivation during disease conditions can lead to detrimental effects on brain function and structure [15].

Cyanide (CN) is a potent, rapid-acting toxin that has significant neurotoxic effects on the brain [16]. It primarily exerts its toxicity through the inhibition of cytochrome c oxidase in the mitochondrial electron transport chain, leading to a disruption of cellular respiration and adenosine triphosphate (ATP) production [17]. This results in cellular hypoxia, metabolic acidosis, and the accumulation of lactate. CN is particularly dangerous in situations where oxygen delivery to tissues is already compromised, such as during ischemic events. In the brain, CN exposure can lead to a cascade of neurophysiological events, including neuronal depolarization, excitotoxicity, and, potentially, irreversible neuronal damage [18,19]. CN-induced neuronal injury has been linked to disturbances in ion homeostasis, including the dysregulation of ion channels [20]. CN’s ability to influence ion channels, such as those involved in neurotransmission and cellular signaling, could significantly alter brain function and exacerbate damage in neurodegenerative conditions or during acute ischemic injury. However, while the mechanisms of CN toxicity are well understood in terms of mitochondrial inhibition and metabolic disruption, its effects on specific ion channels in the brain remain less clear and warrant further investigation.

Previous studies have provided evidence that CN can modulate the activity of ion channels [21], such as N-Methyl-D-Aspartate (NMDA) receptors, which are crucial for synaptic plasticity and memory [22,23]. NMDA receptors are a subtype of glutamate receptors that mediate excitatory neurotransmission and play a key role in synaptic plasticity processes such as long-term potentiation and long-term depression. CN has been shown to enhance NMDA receptor-mediated currents [24], likely through a zinc-dependent mechanism, although the precise pathways remain a subject of ongoing research [25,26,27]. This modulation of NMDA receptors suggests that CN can influence neuronal excitability and synaptic plasticity, which may contribute to its neurotoxic effects.

Given the central role of NMDA receptors in brain function, it is plausible that CN’s action on other ion channels, particularly those involved in pH sensing and neuronal excitability, could also have significant effects on brain activity. One such class of channels, the ASICs, remains understudied in the context of CN exposure. Given that ASICs are activated by extracellular protons and involved in neuronal excitability and acid-base homeostasis, it is important to investigate how CN may influence their function and whether this contributes to its neurotoxic effects. ASICs are integral to the regulation of neuronal excitability, especially in pathological conditions that involve changes in extracellular pH, such as ischemic stroke and traumatic brain injury. Since CN exposure results in metabolic acidosis and a decrease in cellular ATP, it is likely that this could affect the extracellular pH environment and, in turn, influence the activity of ASICs. In particular, the potentiation or inhibition of ASICs by CN could modulate neuronal excitability and exacerbate or mitigate the effects of ischemia or other forms of neurotoxicity.

Although previous studies have demonstrated that CN interacts with other ion channels, such as NMDA receptors [21,24,25,26,27], the potential effects of CN on ASIC channels remain largely unexplored. ASICs, particularly ASIC1a, play a role in excitotoxicity and could be critically involved in CN-induced neuronal injury. Investigating how CN modulates ASIC currents, especially in cortical neurons, is therefore essential for understanding the broader neurotoxic effects of CN and how it influences ion channel function in the brain. The primary hypothesis of the current study is that CN potentiates ASIC currents in cortical neurons through a zinc-dependent mechanism. We hypothesize that CN enhances the sensitivity of ASIC channels, particularly ASIC1a, to extracellular protons, leading to increased neuronal excitability and potentially contributing to CN-induced neurotoxicity. Specifically, we propose that CN enhances ASIC1a currents by binding to a high-affinity zinc site on the channel. This mechanism, involving zinc binding, is known to modulate ASIC channel activity [28]. The potentiation of ASIC currents by CN is likely mediated through this high-affinity zinc-binding site in the extracellular domain of ASIC1a.

To test this hypothesis, we will examine the effects of CN on ASIC currents in cultured mouse cortical neurons, utilizing a combination of pharmacological agents, genetic knockout (KO) models, and electrophysiological techniques. By investigating the mechanisms underlying the CN-induced potentiation of ASIC currents, this study aims to provide novel insights into how CN modulates ion channel function and assess the potential contribution of ASICs to CN-induced neuronal injury.

## 2. Materials and Methods

### 2.1. Primary Cortical Neuronal Cultures

The care and handling of animals in this study were conducted in accordance with the guidelines and protocols approved by the Institutional Animal Care and Use Committee at the University of Missouri–Kansas City. All procedures complied with the ethical standards set forth by the National Institutes of Health guidelines for the use of animals. Every effort was made to minimize animal use and alleviate any discomfort. Primary cultures of mouse cortical neurons were prepared using established methods [28,29]. In brief, time-pregnant BL/6J mice (embryonic day 16), including ASIC1a and ASIC2 knockout (KO) mice, were anesthetized with halothane and euthanized by cervical dislocation. Fetuses were promptly removed and placed in cold Hanks’ solution that lacked calcium and magnesium. The cortex was carefully dissected, incubated with 0.05% trypsin-EDTA for 10 min at 37 °C, and then dissociated by triturating with fire-polished glass pipettes. The resulting cell suspension was plated onto poly-L-ornithine-coated 35 mm culture dishes. Neurons were cultured in Neurobasal medium supplemented with B27 and maintained at 37 °C in a humidified incubator with 5% CO_2_. Cultures were fed twice a week and used for electrophysiological recordings between 10 and 15 days after plating. The ASIC1a and ASIC2 KO mice used in this study were bred at our institution and generously provided by Drs. Wemmie and Welsh from the University of Iowa.

### 2.2. Transient Expression of Functional ASICs in CHO Cells

The tissue culture methods for Chinese Hamster Ovary (CHO) cells (American Type Culture Collection, Manassas, VA, USA) and the protocol for transfecting CHO cells with various ASIC subunits have been previously described in detail [28,29,30]. Briefly, CHO cells were cultured in standard F12 medium (American Type Culture Collection, Manassas, VA, USA) supplemented with 10% fetal bovine serum and incubated at 37 °C in a CO_2_ incubator. Cells were trypsinized with trypsin–EDTA, plated onto 35 mm culture dishes at 10–15% confluence, and allowed to recover for 24 h at 37 °C. Once the cells reached approximately 50–70% confluence, they were transiently transfected with expression vectors containing cDNA for rat ASIC subunits—ASIC1a, ASIC1b, ASIC2a, ASIC2b, and ASIC3—along with enhanced green fluorescent protein at a 1:0.25 molar ratio (Invitrogen, San Diego, CA, USA) using X-tremeGENE HP transfection reagent (Roche Diagnostics, Indianapolis, IN, USA). For co-expression experiments involving ASIC1a and either ASIC2a or ASIC2b, a 2:1 molar ratio of ASIC1a (2) to ASIC2a (1) or ASIC2b (1) was used. Electrophysiological recordings were carried out 48 to 72 h post-transfection. The cDNA clones for rat ASIC1a, ASIC1b, ASIC2a, ASIC2b, and ASIC3 were generously provided by Dr. M. Lazdunski (Institut de Pharmacologie Moléculaire et Cellulaire, Centre National de la Recherche Scientifique, Valbonne, France).

### 2.3. Electrophysiology

Whole-cell voltage-clamp recordings were conducted as previously described [28,29,30]. Patch electrodes, with resistances ranging from 3 to 6 MΩ when filled with intracellular solution, were made from thin-walled borosilicate glass (1.5 mm diameter, WPI, Sarasota, FL, USA) using a two-stage puller (PC-10, Narishige, Tokyo, Japan). Whole-cell ASIC currents were induced by lowering the pH from 7.4 to various levels (e.g., pH 6.5) at a holding potential of −60 mV and recordings were made using Axopatch 200B amplifiers (Axon CNS, Molecular Devices, Foster City, CA, USA). Data were filtered at 2 kHz and digitized at 5 Hz using Digidata 1440 DAC units (Axon CNS, Molecular Devices, Foster City, CA, USA). Data acquisition was performed using pCLAMP software (Version 10.2, Axon CNS, Molecular Devices, Foster City, CA, USA).

Typically, ASIC channels were activated by reducing the pH from 7.4 to the target levels (e.g., pH 6.5) for 4 to 7 s, with recordings taken every 2 min to allow for full recovery from desensitization. A voltage step of −10 mV from the holding potential (−60 mV, unless otherwise noted) was applied periodically during each experiment to monitor cell capacitance and access resistance. Recordings in which the access resistance or capacitance changed by more than 10% during the experiment were excluded from the analysis.

### 2.4. Point Mutagenesis

Site-directed mutagenesis was performed as previously described [28,29,30]. Briefly, the point mutation in ASIC1a was introduced using the Quick-Change Site-Directed Mutagenesis system (Stratagene, La Jolla, CA, USA), according to the manufacturer’s instructions. The primers for mutagenesis were obtained from Sigma-Genosys (The Woodlands, TX, USA). The mutation at lysine 133 in ASIC1a was confirmed through restriction enzyme digestion and DNA sequencing. To ensure precision, the entire rat ASIC1a cDNA was sequenced to verify that no unintended mutations were introduced.

### 2.5. Solutions and Compounds

Standard extracellular fluid (ECF) contained (mM) 140 NaCl, 5.4 KCl, 2.0 CaCl_2_, 1.0 MgCl_2_, 20 HEPES, and 10 glucose (pH 7.4; 320~330 mOsm). For solutions with a pH of 6.0 or lower, MES was used instead of HEPES for more reliable pH buffering [28,29,30]. The pipette solution contained (mM) 140 K-Gluconate, 10 HEPES, 11 EGTA, 2 TEA, 1 CaCl_2_, 2 MgCl_2_, and 4 K_2_ATP (pH 7.2~7.3; 290~300 mOsm). All chemicals were purchased from Sigma-Aldrich (St. Louis, MO, USA). A multi-barrel perfusion system (SF-77, Hamden, CT, USA) was employed to achieve a rapid exchange of extracellular solutions. For the pretreatment with cyanide protocol, cyanide was present in the ECF of both pH 7.4 and lower pH (e.g., 6.5). This method was described in our previous publications [28,29,30].

### 2.6. Data Analysis

All data were analyzed using Clampfit 10.2 software (Axon CNS, Molecular Devices, Foster City, CA, USA). For experiments involving varying concentrations of CN (0.01, 0.03, 0.1, 0.3, 1.0, 3.0, and 10.0 mM), ASIC currents were normalized to control values obtained in the absence of CN treatment.

For pH activation curves, the ECF from one barrel of the perfusion system was maintained at pH 7.4, while the ECF from the second barrel was sequentially switched to pH values of 7.0, 6.8, 6.5, 6.0, 5.0, and 4.0 using the SF-77B fast perfusion system (Warner Instrument Co., CT, USA). The acid-induced currents at each pH were normalized to the peak current measured at pH 4.0. These normalized values were then fitted to the Hill equation using SigmaPlot 10 software to determine the pH50 values (the pH at which the current reached half of its maximum). To evaluate the sustained component of the ASIC currents, measurements were taken 6 s after the pH drop.

### 2.7. Statistics

Statistical analyses were conducted using SigmaPlot software. Data were presented as the mean ± standard error of the mean (SEM) for each experimental subgroup. Significant differences between mean values of the experimental groups were evaluated using a Student’s *t*-test for two-group comparisons and a one-way analysis of variance (ANOVA) for multiple pairwise comparisons, with post-hoc testing performed using the Bonferroni method. Differences were considered statistically significant when *p* < 0.05.

## 3. Results

### 3.1. Rapid Potentiation of ASIC Currents by CN on Cortical Neurons

**Figure 1:**
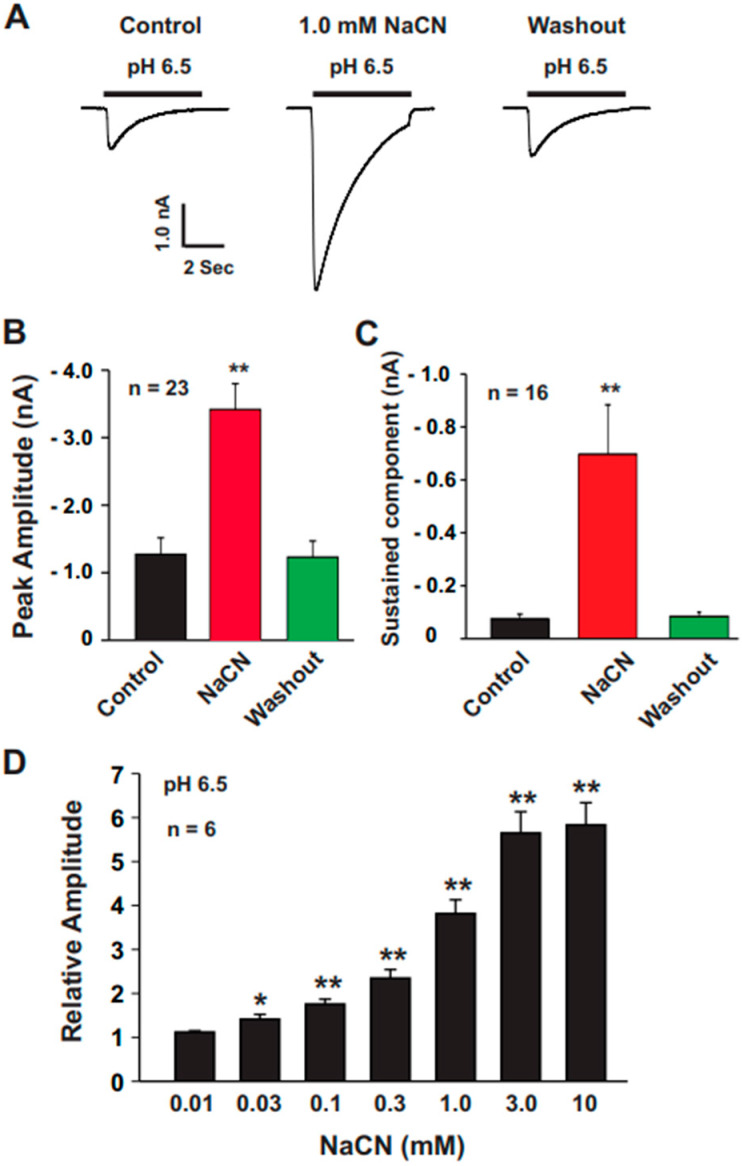
Rapid potentiation of ASIC currents by CN in cultured mouse cortical neurons. (**A**) Representative traces showing the effect of CN (1.0 mM) on ASIC currents triggered by a pH drop from 7.4 to 6.5, recorded from cultured mouse cortical neurons. The traces include control, CN treatment, and washout conditions. (**B**) Statistical analysis indicating that CN (1.0 mM) significantly enhanced the peak amplitude of ASIC currents (n = 23, ** p < 0.01). The CN group was compared with the control and washout groups. (**C**) Statistical data showing that CN (1.0 mM) also significantly increased the sustained component of ASIC currents (n = 16, ** p < 0.01). The CN group was compared with the control and washout groups. (**D**) Dose–response data revealing that CN potentiated ASIC currents in a dose-dependent manner, with an EC_50_ of 0.85 ± 0.02 mM (n = 6; comparisons made to CN at 0.01 mM; * p < 0.05; ** p < 0.01).

**Figure 2:**
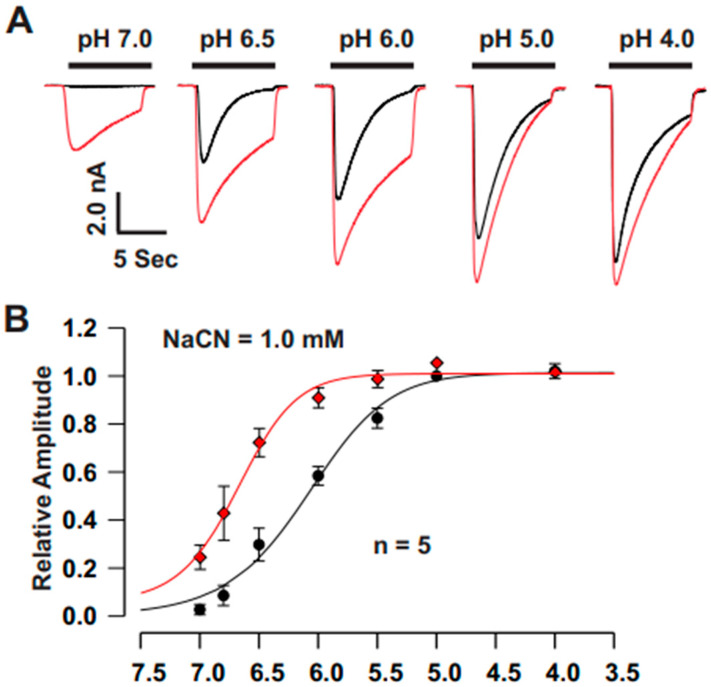
CN treatment induces a leftward shift in the pH dose–response curve in cultured mouse cortical neurons. (**A**) Representative traces showing the effect of CN (1.0 mM) on ASIC currents triggered by pH drops from 7.4 to 7.0, 6.8, 6.5, 6.0, 5.0, and 4.0 in cultured mouse cortical neurons. (**B**) pH dose–response curves with (red line) and without (black line) CN treatment. CN (1.0 mM) caused a leftward shift in the pH dose–response curve, changing the half-maximal pH (pH_50_) from 6.68 ± 0.03 to 6.09 ± 0.06 (n = 5). The peak amplitude of ASIC currents was measured and the data are presented as the mean ± SEM.

Functional ASICs are highly expressed in mouse cortical neurons [28,29]. To investigate the effect of CN on ASIC currents, we recorded ASIC currents from cultured mouse cortical neurons. CN (1.0 mM) alone did not elicit any detectable currents. As shown in Figure 1A–C, acute pretreatment with CN for 2 min at concentrations of 1.0 mM significantly potentiated both the peak amplitude and sustained component of ASIC currents in 23 cortical neurons tested. The potentiation of ASIC currents by CN was rapidly reversed after a 2-min washout. We further explored the dose–response relationship of CN on ASIC currents. As shown in Figure 1D, CN dose-dependently potentiated the ASIC currents in cortical neurons with an EC_50_ of 0.85 ± 0.02 mM. Additionally, we examined the pH dose–response curve with and without CN treatment (Figure 2A). CN (1.0 mM) treatment caused a significant leftward shift of pH dose–response curve, changing the half-maximal pH (pH_50_) from 6.09 ± 0.03 to 6.68 ± 0.03, as shown in Figure 2B. These results suggest that CN potentiates ASIC currents in a concentration-dependent manner and modulates the pH sensitivity of ASIC channels.

### 3.2. Prolonged CN Treatment Did Not Further Increase the Potentiation of ASIC Currents

**Figure 3:**
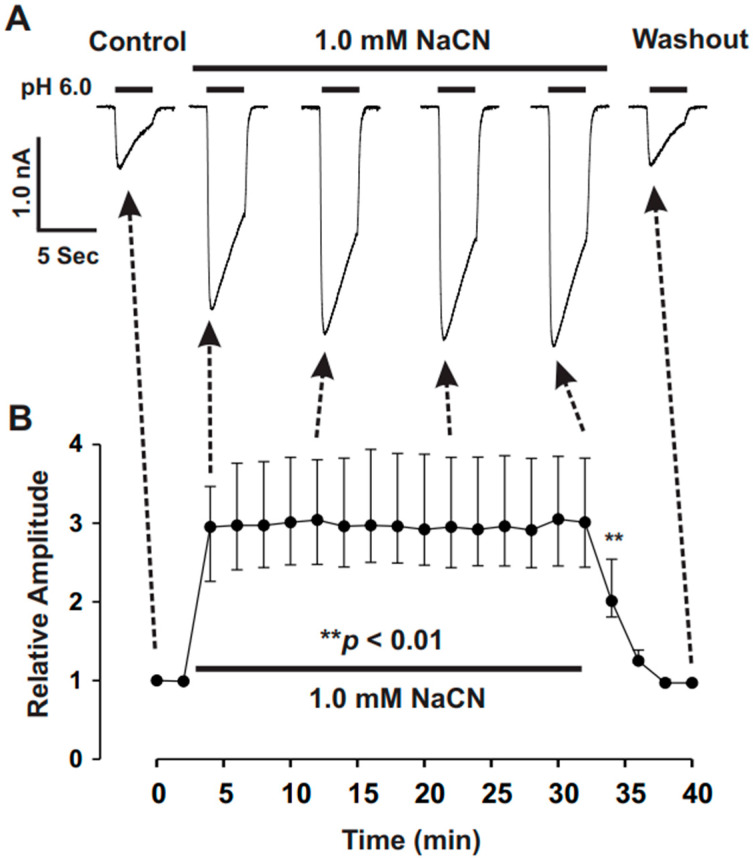
Prolonged treatment with CN does not further enhance the potentiation of ASIC currents in cultured mouse cortical neurons. (**A**) Representative traces showing the effect of CN (1.0 mM) on ASIC currents triggered by a pH drop from 7.4 to 6.0 over different time periods. The traces include control, CN treatment (30 min), and washout conditions. (**B**) Statistical analysis of ASIC peak amplitude, indicating that CN (1.0 mM) significantly enhanced the peak amplitude of ASIC currents within 30 min of treatment, with no further potentiation observed beyond the first 2 min of treatment (n = 5, ** *p* < 0.01). The CN group was compared with the control and washout groups.

Our previous findings indicated that brief treatments with redox reagents, such as dithiothreitol (DTT), potentiated ASIC currents and produced a long-lasting response [29]. However, in the present study, the potentiation of ASICs by CN was observed to reverse quickly (Figure 1A). We next sought to determine whether prolonged exposure to CN would have additional effects on ASIC currents. As shown in Figure 3, CN treatment led to an increase in ASIC currents within the first 2 min, but further treatment over 30 min did not produce any additional potentiation of peak amplitude. These results suggest that the potentiation of ASIC currents by CN does not require extended treatment, which may otherwise influence intracellular ATP synthesis.

### 3.3. The Absence of Intracellular ATP Treatment Did Not Reveal Effects on ASIC Potentiation by CN

**Figure 4:**
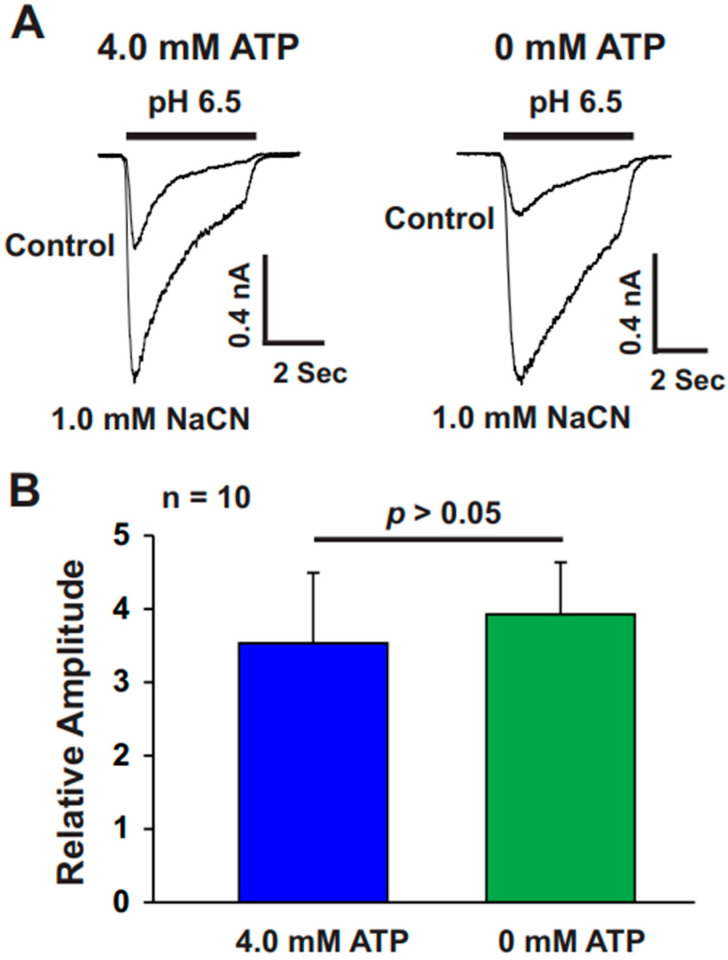
The potentiating effect of CN on ASIC currents is independent of intracellular ATP levels. (**A**) Representative traces showing the potentiation of ASIC currents by CN with 4.0 mM ATP in the recording pipette (left panel) and without ATP in the pipette (right panel). (**B**) Statistical analysis of ASIC peak amplitudes indicating no significant difference between conditions with 4.0 mM ATP and without ATP in the pipette (n = 10, *p* > 0.05). The peak amplitude of ASIC currents in the 4.0 mM ATP group was compared to the peak amplitude of ASIC currents in the 0 mM ATP group in the recording pipette.

A previous study has shown that CN inhibits the respiratory chain, thereby disrupting ATP synthesis [17]. To further investigate the effect of intracellular ATP on the CN-induced potentiation of ASIC currents, we recorded ASIC currents from two groups of cultured mouse cortical neurons, either in the presence or absence of 4.0 mM ATP in the recording pipette. In the group with ATP in the pipette, recordings were initiated 10 min after achieving the whole-cell seal to ensure equilibrium between the pipette and cytosolic ATP levels. As shown in Figure 4A (left panel), CN treatment significantly enhanced ASIC currents in the presence of 4.0 mM pipette ATP. In a separate experiment, ASIC currents were recorded without ATP in the pipette (Figure 4A, right panel). We found that CN treatment also potentiated ASIC currents in this group. Importantly, there was no significant difference in the potentiation of ASIC currents between the two conditions (with or without ATP in the pipette), as shown in Figure 4B. These results suggest that the potentiating effect of CN on ASIC currents is independent of intracellular ATP levels.

### 3.4. TPEN and CN Had a Similar Effect in Potentiating the ASIC Current

**Figure 5:**
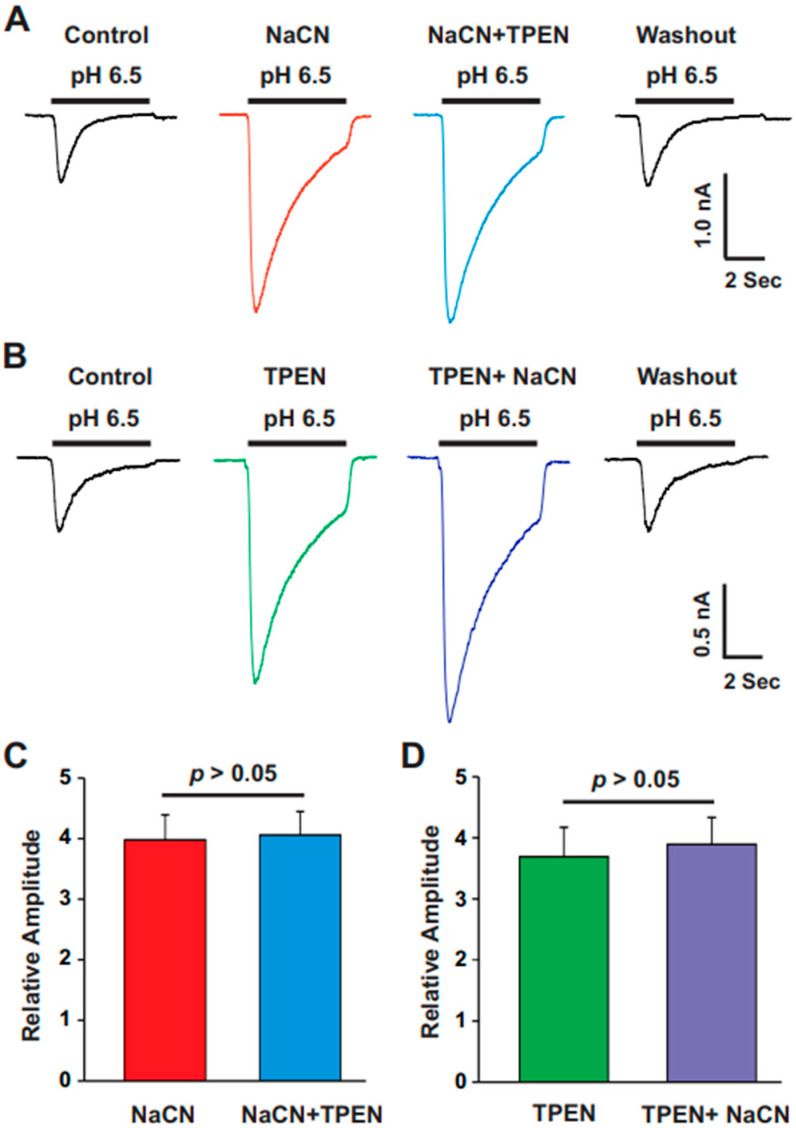
TPEN and CN exhibited a similar effect in potentiating the ASIC current. (**A**) Representative traces showing the effect of CN (1.0 mM) and CN (1.0 mM) + TPEN (10 µM) on ASIC currents triggered by a pH drop from 7.4 to 6.5 in cultured mouse cortical neurons. CN treatment was applied first, followed by CN with TPEN, with each treatment lasting 2 min. (**B**) Representative traces showing the effect of TPEN (10 µM) and CN (1.0 mM) + TPEN (10 µM) on ASIC currents triggered by a pH drop from 7.4 to 6.5 in cultured mouse cortical neurons. TPEN treatment was applied first, followed by TPEN with CN, with each treatment lasting 2 min. (**C**,**D**) Statistical analysis of ASIC peak amplitude showing no significant difference between the conditions with CN and CN + TPEN or TPEN and TPEN + CN (n = 10, *p* > 0.05). In panel (**C**), the CN group is compared to the CN plus TPEN group, while, in panel (**D**), the TPEN group is compared to the TPEN plus CN group.

High-affinity zinc refers to zinc that binds strongly to its target molecules or receptors, even at low concentrations, typically in the nanomolar (nM) range. This type of zinc binds tightly to receptors or proteins that require only minimal amounts of zinc to exert a biological effect, meaning that even low levels (nM) can trigger a response [25]. High-affinity zinc binding to ion channels and receptors has a broad and significant impact on cellular function, particularly within the nervous system [26]. It modulates neurotransmitter signaling, ion flow, and receptor activity, influencing processes like synaptic plasticity, neuroprotection, and cellular signaling [27]. The precise regulation of zinc binding is crucial for maintaining the balance between excitatory and inhibitory neurotransmission. Disruptions in this balance are linked to various neurological and psychiatric disorders. Our previous study demonstrated that the high-affinity zinc chelator N,N,N’,N’-Tetrakis(2-pyridylmethyl) ethylenediamine (TPEN) significantly potentiated ASIC currents in an ASIC1a-dependent manner [28]. In this experiment, we aimed to determine whether CN has a similar effect to TPEN. To do this, we recorded ASIC currents under different treatment protocols. First, we recorded ASIC currents with CN (1.0 mM), which enhanced the currents. After the response stabilized with CN, we added TPEN (10 µM) together with CN (1.0 mM), but no further change in the ASIC currents was observed (Figure 5A,C). In a separate experiment, we applied TPEN treatment (10 µM) first, which also potentiated the ASIC currents, and subsequently added CN (1.0 mM) together with TPEN (10 µM); however, CN (1.0 mM) did not further enhance the ASIC currents (Figure 5B,D). Collectively, these data suggest that the potentiation of ASIC currents by CN and TPEN occurs through similar mechanisms, as the effects of one did not enhance the actions of the other.

### 3.5. Low-Affinity Zinc Blocked the Potentiation of ASIC Currents by CN

**Figure 6:**
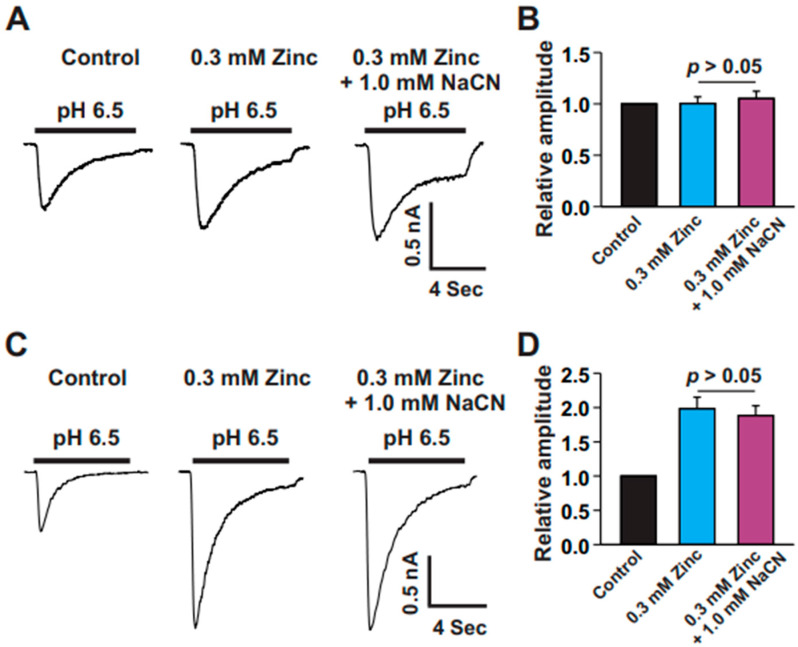
Low-affinity zinc inhibited the potentiation of ASIC currents by CN. (**A**) Representative traces illustrating the effect of zinc (0.3 mM) and zinc (0.3 mM) + CN (1.0 mM) on ASIC currents induced by pH drops from 7.4 to 6.5 in cultured mouse cortical neurons. Zinc was applied first and had no effect on the ASIC current, followed by the combination of zinc and CN, with each treatment lasting 2 min. (**B**) Statistical analysis of ASIC peak amplitude, showing no significant difference between zinc and zinc + CN conditions (n = 8, *p* > 0.05). (**C**) Representative traces showing that zinc (0.3 mM) potentiated the ASIC current, while zinc (0.3 mM) + CN (1.0 mM) did not induce further potentiation of ASIC currents triggered by pH drops from 7.4 to 6.5 in cultured mouse cortical neurons. Zinc was applied first, followed by zinc + CN, with each treatment lasting 2 min. (**D**) Statistical analysis of ASIC peak amplitude showing no significant difference between zinc and zinc + CN conditions (n = 7, *p* > 0.05).

Low-affinity zinc refers to zinc that binds more weakly to its targets at higher concentrations, typically in the micromolar (μM) range [30,31,32,33,34]. Unlike high-affinity binding, where small amounts of zinc can significantly influence cellular functions [28], low-affinity zinc requires larger amounts to trigger a response, and its binding is more easily reversible [30,31,32,33,34]. Our previous study demonstrated that low-affinity zinc (0.3 mM) either enhances or has no significant effect on ASIC currents in cortical and striatal neurons [28,31]. To investigate whether low-affinity zinc influences the effect of CN on cortical neurons, we recorded ASIC currents following a pH drop to 6.5. We identified two types of neurons with ASIC currents in cultured mouse cortical neurons. In one type, a 0.3 mM concentration of zinc had no effect on the ASIC currents, while, in the other type, the same concentration of zinc enhanced the ASIC currents. In the first set of experiments, we applied 0.3 mM zinc to examine its effect on ASIC currents. As shown in Figure 6A,B, 0.3 mM zinc had no impact on the ASIC currents. We then co-applied 0.3 mM zinc with 1.0 mM CN and found that this combination did not further enhance the current amplitude. In a separate set of experiments, when 0.3 mM zinc was applied first (see Figure 6C,D), it potentiated the ASIC currents. However, subsequent treatment with 1.0 mM CN plus 0.3 mM zinc did not produce any additional potentiation. Taken together, these results suggest that low-affinity zinc inhibits the potentiating effects of high-affinity zinc through CN.

### 3.6. CN Potentiated the ASIC Currents in Cortical Neurons from ASIC2 but Not ASIC1a KO Mice

**Figure 7:**
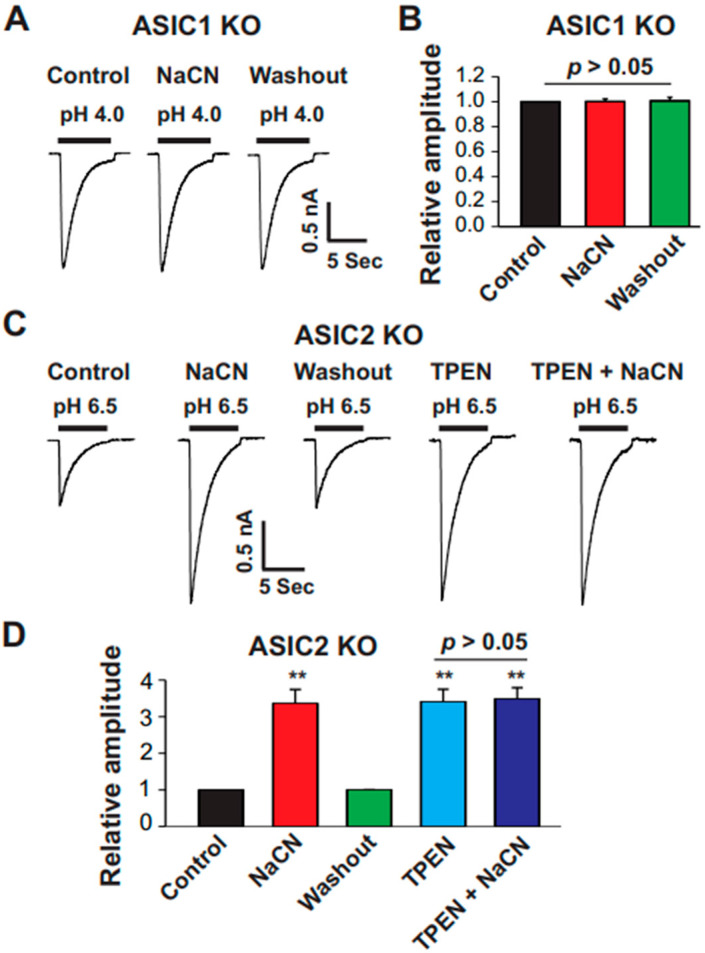
CN potentiated ASIC currents in cultured mouse cortical neurons from ASIC2 but not from ASIC1a KO mice. (**A**) Representative traces showing the effect of CN (1.0 mM) on ASIC currents triggered by pH drops from 7.4 to 4.0 in cultured mouse cortical neurons from ASIC1a KO mice. (**B**) Statistical analysis of ASIC peak amplitude showing no significant difference between the conditions with control, CN, or washout groups (n = 10, *p* > 0.05). The CN group was compared with the control and washout groups. (**C**) Representative traces showing the effect of CN (1.0 mM), TPEN (10 µM), and TPEN (10 µM) + CN (1.0 mM) on ASIC currents triggered by pH drops from 7.4 to 6.5 in cultured mouse cortical neurons from ASIC2 KO mice. CN treatment was applied first, followed by washout, treatment with TPEN, and then treatment by TPEN with CN, with each treatment lasting 2 min. (**D**) Statistical analysis of ASIC peak amplitude, showing a significant difference between the conditions with CN and control, TPEN and control, and TPEN + CN and control (n = 10, ** p < 0.01), but no significant difference between TPEN and TPEN with CN (n = 10, *p* > 0.05). The CN group was compared with the control and washout groups. The TPEN group was compared to the TPEN plus CN group.

To determine whether the ASIC1a or ASIC2 subunit was involved in the potentiation by CN, we recorded ASIC currents from cultured cortical neurons of ASIC1a and ASIC2 KO mice. No detectable ASIC currents were recorded from neurons of the ASIC1a KO mice at pH 6.0. After lowering the pH to 4.0, ASIC currents were observed in some neurons, but CN (1.0 mM) did not potentiate these currents. In contrast, when we recorded ASIC currents from neurons of the ASIC2 KO mouse at pH 6.5, both CN (1.0 mM) and TPEN (10 µM) treatment potentiated all recorded currents; when added together, no further potentiation was observed (Figure 7C,D). These results suggest that ASIC1a may play a critical role in the potentiation of ASIC currents by CN, while ASIC2 appears to be dispensable for this effect.

### 3.7. Homomeric ASIC1a and Heteromeric ASIC1a/2, but Not ASIC1b, ASIC2a, and ASIC3, Are Targets of CN

**Figure 8:**
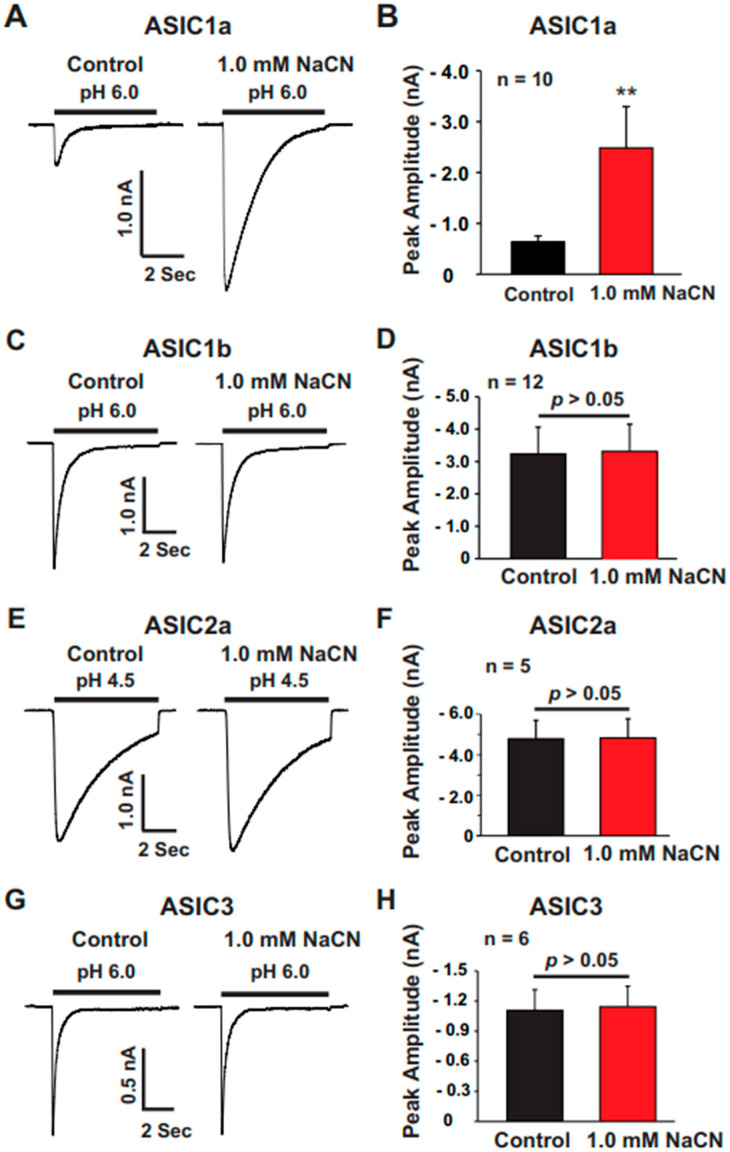
ASIC1a-dependent potentiation of ASIC currents in CHO cells expressing different functional ASIC subunits. (**A**) Representative traces illustrating the effect of CN (1.0 mM) on ASIC currents triggered by pH drops from 7.4 to 6.0 in CHO cells expressing ASIC1a. (**B**) Statistical analysis of ASIC1a peak amplitude showing a significant difference between CN-treated and control conditions (n = 10, ** *p* < 0.01). (**C**) Representative traces demonstrating the effect of CN (1.0 mM) on ASIC currents triggered by pH drops from 7.4 to 6.0 in CHO cells expressing ASIC1b. (**D**) Statistical analysis of ASIC1b peak amplitude revealing no significant difference between CN-treated and control conditions (n = 12, *p* > 0.05). (**E**) Representative traces showing the effect of CN (1.0 mM) on ASIC currents triggered by pH drops from 7.4 to 4.5 in CHO cells expressing ASIC2a. (**F**) Statistical analysis of ASIC2a peak amplitude indicating no significant difference between CN-treated and control conditions (n = 5, *p* > 0.05). (**G**) Representative traces depicting the effect of CN (1.0 mM) on ASIC currents triggered by pH drops from 7.4 to 6.0 in CHO cells expressing ASIC3. (**H**) Statistical analysis of ASIC3 peak amplitude showing no significant difference between CN-treated and control conditions (n = 6, *p* > 0.05). The CN group is compared with the control group in (**B**,**D**,**F**,**H**).

**Figure 9:**
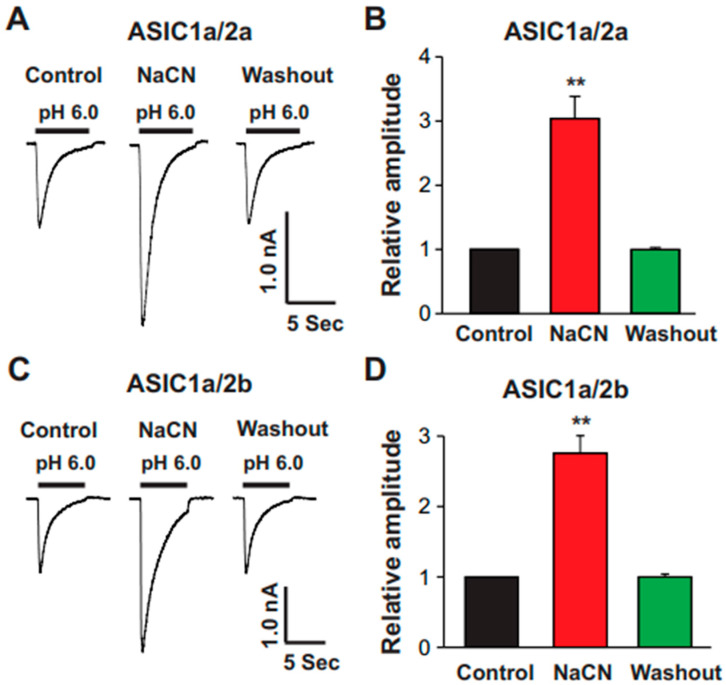
Potentiation of heteromeric ASIC1a/2a and ASIC1a/2b by CN. (**A**) Representative traces showing the effect of CN (1.0 mM) on ASIC currents triggered by pH drops from 7.4 to 6.0 in CHO cells expressing ASIC1a/2a. (**B**) Statistical analysis of ASIC1a/2a peak amplitude indicating a significant difference between CN-treated and control conditions (n = 6, ** p < 0.01). The CN group was compared with the control and washout groups. (**C**) Representative traces showing the effect of CN (1.0 mM) on ASIC currents triggered by pH drops from 7.4 to 6.0 in CHO cells expressing ASIC1a/2b. (**D**) Statistical analysis of ASIC1a/2b peak amplitude demonstrating a significant difference between CN-treated and control conditions (n = 5, ** p < 0.01). The CN group was compared with the control and washout groups.

The brain cortex expresses various ASIC subunits [28,29]. To identify which ASIC subunit is involved in the potentiation of ASIC currents by CN, we expressed functional ASIC subunits—ASIC1a, ASIC1b, ASIC2a, and ASIC3—in CHO cells, which do not contain endogenous ASICs (note: ASIC2b and ASIC4 cannot form functional channels on their own). As shown in Figure 8, CN at a concentration of 1.0 mM potentiated the ASIC currents only in cells expressing ASIC1a (Figure 8A,B) but not in those expressing ASIC1b (Figure 8C,D), ASIC2a (Figure 8E,F), or ASIC3 (Figure 8G,H). Furthermore, when we co-expressed ASIC1a with either ASIC2a or ASIC2b, CN (1.0 mM) potentiated the ASIC1a/2a (Figure 9A,B) and ASIC1a/2b (Figure 9C,D) heteromeric channels. These data suggest that the potentiation of ASIC currents by CN specifically requires the presence of ASIC1a subunits in the heteromeric channels, indicating a subunit-specific mechanism of potentiation.

### 3.8. High-Affinity Zinc-Binding Site K133 in the Extracellular Domain of ASIC1a Is Responsible for CN Potentiation

**Figure 10:**
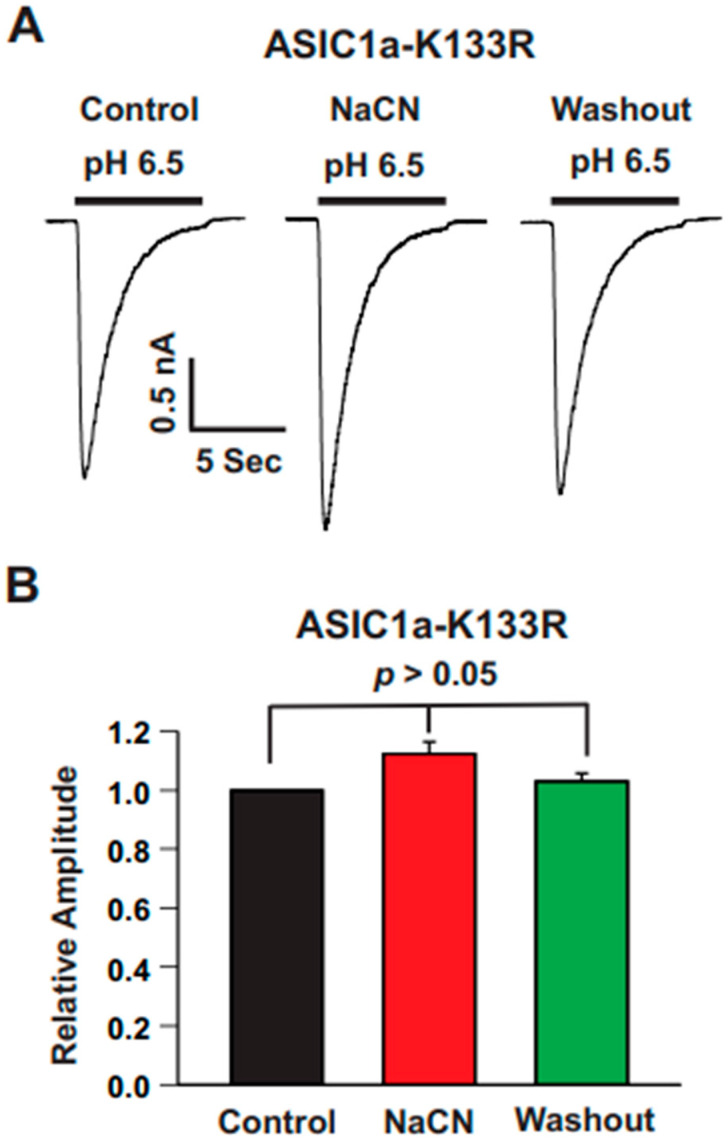
High-affinity zinc-binding site K133 in the extracellular domain of ASIC1a is responsible for CN potentiation. (**A**) Representative traces showing the effect of CN (1.0 mM) on ASIC currents triggered by pH drops from 7.4 to 6.5 in CHO cells expressing ASIC1a-K133R. (**B**) Statistical analysis of ASIC1a-K133R peak amplitude indicating no significant difference between CN-treated and control conditions (n = 8, *p* > 0.05). The CN group was compared with the control and washout groups.

Our previous studies have shown that TPEN, a zinc chelator, potentiates ASIC1a currents and that lysine 133 in the extracellular domain of ASIC1a plays a key role in this effect [28]. First, we analyzed the pH–dose response curve for ASIC1a-K133R, which revealed a pH_50_ of 6.28 ± 0.03 (n = 5), similar to that of wild-type ASIC1a (pH_50_ of 6.21 ± 0.02; n = 5). To investigate whether CN affected ASIC1a in a similar manner, we then tested its effect on the ASIC1a-K133R mutant. As shown in Figure 10, CN at a concentration of 1.0 mM had no effect on ASIC1a-K133R currents. These results suggest that CN potentiates ASIC1a currents through a mechanism involving the interaction with lysine 133.

## 4. Discussion

The present study provides significant insights into the effect of CN on ASICs in cultured mouse cortical neurons, with a particular focus on the potentiation of ASIC currents by CN. The key findings of this study are as follows: (1). We found that CN rapidly potentiated the ASIC currents in cultured mouse cortical neurons in a dose-dependent manner, shifting the pH dose–response curve to the left. This suggests that CN enhances the sensitivity of ASICs to changes in extracellular pH. (2). The potentiation of ASIC currents by CN was observed regardless of whether ATP was present in the recording pipette or not. The results indicate that the perturbation of intracellular ATP levels was not involved in the CN-mediated potentiation of ASIC currents, although the main target of CN is mitochondrial respiration. (3). We further revealed that the potentiation effect of CN on ASIC currents was tightly linked to the interaction with zinc. Specifically, CN was shown to bind with high affinity to zinc, and this binding was essential for the potentiation of ASIC currents. The use of a high-affinity zinc chelator, TPEN, blocked the potentiation of ASIC currents by CN, while treatment with low-affinity zinc had no such effect, supporting the critical role of zinc in this process. (4). We also explored the specificity of CN’s effect on different ASIC subtypes. CN potentiated ASIC currents in neurons from ASIC2 KO mice but not from ASIC1a KO mice. Additionally, CN had no effect on ASIC1b, 2a, or 3 channels but potentiated currents from homomeric ASIC1a and heteromeric ASIC1a/2 channels. This indicates that CN’s potentiating effect is specific to certain ASIC subtypes, particularly ASIC1a. (5). We identified lysine 133 (K133) in the extracellular domain of the ASIC1a subunit as a key residue responsible for the potentiation effect of CN. The mutation of K133 to arginine (K133R) abolished the CN-induced potentiation of ASIC1a currents, further reinforcing the importance of this residue in CN’s mechanism of action.

The findings of this study highlight the unique interaction between CN and ASIC channels, particularly ASIC1a, and provide a deeper understanding of how CN can modulate ion channel activity in the brain. The potentiation of ASIC currents by CN is explained primarily through its high-affinity interaction with zinc, a metal ion known to modulate the activity of ion channels. CN is a potent, rapid-acting toxin, and its effect on ASIC currents is mediated by its interaction with zinc. In the present study, we found that TPEN (10 nM), a high-affinity zinc chelator, potentiated ASIC currents, and CN at a concentration of 1.0 mM also potentiated ASIC currents in the same neuron to a similar extent. However, when TPEN and CN were applied together, the potentiation was similar to that observed with either TPEN or CN alone; thus, our data suggest that TPEN and CN likely act through similar mechanisms to potentiate ASIC currents. Zinc is known to act as a potent modulator of ASICs, particularly in the extracellular domain of ASIC1a, which is rich in histidine and cysteine residues that can interact with metal ions [25,26]. The presence of high-affinity zinc-binding sites on ASICs, particularly at the K133 residue in ASIC1a, is crucial for this modulation [28]. The observation that CN potentiates ASIC currents in neurons from ASIC2 KO mice but not from ASIC1a KO mice suggests that ASIC1a is the primary target for CN-induced potentiation. The results from CHO cells expressing homomeric and heteromeric ASIC subtypes also support this conclusion, showing that CN potentiates currents specifically in ASIC1a and ASIC1a/2 channels and not in ASIC1b, 2a, or 3 channels. This specificity likely arises from structural differences in the extracellular domains of these channels, which may impact their ability to interact with zinc and other modulators such as CN. The mutation of K133 to arginine in the extracellular domain of ASIC1a provides crucial insight into the mechanism of CN’s action. K133 is located in a region that is known to interact with zinc, and its mutation disrupts the high-affinity binding of zinc to the ASIC1a subunit, thereby preventing the potentiation of ASIC1a currents by CN. This suggests that the K133 residue is a key structural feature that mediates CN’s effect on ASIC1a and likely contributes to the specificity of CN’s action on this channel subtype. While lysine and zinc would normally repel each other due to their positive charges, other factors such as the structural context of the ASIC1a subunit, coordination with other amino acid residues, or the divalent nature of zinc might allow for their interaction. The mutation of lysine 133 likely disrupts this delicate interaction, which is why it blocks the high-affinity zinc inhibition of the ASIC1a current. Further studies are needed to clarify the exact mechanism of interaction between zinc and lysine at the extracellular site of the ASIC1a channel.

The findings of this study hold significant implications for our understanding of how CN affects neuronal ion channels and contribute to the broader field of neurotoxicity and neurodegeneration. CN is a well-known toxicant, and its rapid action on the nervous system is often linked to its ability to disrupt cellular respiration and energy metabolism. However, this study adds another layer of complexity by showing that CN can also directly modulate ion channel activity, particularly ASICs. By potentiating ASIC currents, CN may exacerbate neuronal excitability and contribute to neurotoxic effects. This mechanism may be particularly relevant in the context of acute CN poisoning, where rapid neuronal depolarization could lead to excitotoxicity and neuronal damage. ASICs are involved in a range of neurological and psychological disorders, including ischemia [6,7], epilepsy [10,11], and chronic pain [12,13]. By modulating ASIC activity, CN may influence the pathophysiology of these conditions. Understanding how CN interacts with ASICs could open new avenues for therapeutic interventions. For example, targeting the interaction between CN and zinc could provide strategies for mitigating the neurotoxic effects of CN exposure. The identification of the K133 residue as a critical site for the CN-induced potentiation of ASIC1a currents could inform the development of drugs that specifically modulate ASIC1a activity. This could be particularly valuable in conditions where ASICs play a pathological role, such as in ischemic stroke or neurodegenerative diseases. The ability to selectively block or enhance ASIC1a activity could have therapeutic benefits for a variety of neurological disorders.

The current study lays the foundation for several important directions in future research. While this study focused on the interaction between CN and ASICs, the role of zinc in neurotoxicity and neurodegeneration warrants further exploration. Zinc dysregulation is implicated in a variety of neurological conditions [35,36], including Alzheimer’s disease, Parkinson’s disease, and amyotrophic lateral sclerosis. Future studies could investigate how other environmental toxins or physiological conditions that alter zinc homeostasis might affect ASIC function and contribute to disease progression. This study focused on cortical neurons, but CN’s effects on other neuronal subtypes and brain regions remain to be explored. Investigating whether CN has similar effects on ASIC channels in different parts of the brain, such as the hippocampus or basal ganglia, could provide valuable insights into how CN affects brain function more broadly. The identification of K133 as a critical residue in CN-induced potentiation opens the door for the design of small molecules or peptide inhibitors that could block the interaction between CN and ASIC1a. These compounds could be tested for their ability to mitigate CN toxicity and could serve as potential therapeutics for poisoning or other conditions involving excessive ASIC1a activity. While this study focused on acute exposure to CN, it would be interesting to examine the long-term effects of chronic or sub-lethal CN exposure on ASIC function and neuronal health. The chronic modulation of ASIC activity could have lasting consequences on neuronal excitability, synaptic plasticity, and brain function, which should be explored in future studies. Finally, further studies in animal models of CN toxicity would be valuable to confirm the findings observed in cultured neurons. In vivo studies could help elucidate the physiological and behavioral consequences of the CN-induced potentiation of ASIC currents, providing a more comprehensive understanding of CN’s neurotoxic effects.

## 5. Conclusions

In summary, the findings of this study provide critical insights into the interaction between CN and ASICs, particularly ASIC1a. The potentiation of ASIC currents by CN, mediated through its interaction with zinc and the K133 residue in the ASIC1a subunit, represents a novel mechanism by which CN may influence neuronal function. These findings have significant implications for our understanding of CN toxicity and its potential role in neurological diseases. Future research will be essential to further investigate the broader implications of these findings and explore therapeutic strategies for modulating ASIC function in the context of CN exposure and other neurological disorders.

## Data Availability

The datasets in this work are from a public database. All data generated during this study are included in the manuscript.

## Abbreviations

The following abbreviations are used in this manuscript:

ASICs	Acid-sensing ion channels
ATP	Adenosine triphosphate
CHO	Chinese hamster ovary
CN	Cyanide
ECF	Extracellular fluid
KO	Knockout
NMDA	N-Methyl-D-Aspartate
TPEN	N,N,N’,N’-Tetrakis(2-pyridylmethyl)ethylenediamine
